# Cognitive stimulation in socioeconomically disadvantaged children with neurodevelopmental disorders: a case series

**DOI:** 10.3389/fpsyg.2024.1365697

**Published:** 2024-07-09

**Authors:** Pablo Rodríguez-Prieto, Nathalia Alejandra Giral-Oliveros, Ian Craig Simpson, Joaquín A. Ibáñez-Alfonso

**Affiliations:** ^1^Human Neuroscience Laboratory, Department of Psychology, Universidad Loyola Andalucía, Seville, Spain; ^2^Instituto de Desarrollo de la Universidad Loyola Andalucía, Fundación ETEA, Córdoba, Spain; ^3^Department of Experimental Psychology, Universidad de Granada, Granada, Spain

**Keywords:** neuropsychology, cognitive stimulation, neurodevelopmental disorders, vulnerability, socioeconomic level, child development

## Abstract

**Introduction:**

Research shows how conditions in socio-economically disadvantaged environments can be a risk factor for children’s cognitive development. Consequently, children with neurodevelopmental disorders growing up in such environments face a double challenge. This study analyzed the effect of a comprehensive cognitive stimulation program on 4 single case studies comprising children with neurodevelopmental disorders from Guatemala.

**Methodology:**

A descriptive study was conducted, using a case series approach, consisting of four participants with neurodevelopmental disorders, and a neurotypical group of 126 children. Participants in the neurotypical group were randomly assigned to either a control or experimental reference group. Cognitive assessments were performed pre- and post-intervention for all participants. Children in the experimental group received a comprehensive cognitive stimulation program between assessments. Two participants with neurodevelopmental disorders also received the stimulation program while the other two children with neurodevelopmental disorders performed the same task as the control group, specifically, regular reading activities.

**Results:**

The experimental group exhibited a significant improvement in executive functions (inhibition, flexibility, and planning). The two experimental group children with neurodevelopmental disorders exhibited improved social cognition, showing a larger improvement compared to neurotypical children in their group, as well as compared to the two control children. However, although the reading program improved the language skills of the neurotypical control group, the children with neurodevelopmental disorders did not show as much improvement.

**Conclusion:**

These results suggest that specialized interventions are beneficial for children from socio-economically disadvantaged backgrounds, but importantly, may have a larger impact on children with neurodevelopmental disorders.

## 1 Introduction

According to the United Nations, one in five children in the world lives in extreme poverty and the severe negative effects of these conditions can have a significant impact throughout their lives (United Nations, 2020). Guatemala is one of the most diverse nations in Central America, with one of the highest rates of poverty and social inequality in the world. Situations of violence, food insecurity, and discrimination, combined with the social exclusion of indigenous groups, the unstable political structure, and the lack of access to justice, affect the Guatemalan population in a multidimensional way. This causes, among other things, serious alterations in the physical and mental health of children and adolescents who grow up in this environment (Velásquez, 2022).

Previous research has shown that growing up in low socio-economic environments, determined by deprivation of goods and services, occupation, parental education, and low income, as well as exposure to biological and environmental risk factors, has negative physiological and psychological effects on children’s development (Baker, 2014; Company-Córdoba et al., 2020, 2022; Ibáñez-Alfonso et al., 2021). These conditions can lead to modifications in brain structures and functions (Baker-Henningham and López Boo, 2014), altering the development of cognitive processes such as language (Arán-Filippetti, 2012), memory (Johnson et al., 2016), or executive functions (Azar et al., 2019). In addition, growing up in these conditions is associated with psychological and social difficulties in adolescence and adulthood, related to low intellectual achievement, poor academic performance, and limited economic opportunities, and all of these combine to perpetuate social inequalities (Heckman and Masterov, 2007).

Given the importance and impact of the environment on brain maturation, several studies have correlated socioeconomic variables with the presence of neurodevelopmental disorders (NDD). Although this may vary depending on the disorder and geographical location, some studies suggest that children growing up in disadvantaged socioeconomic environments are more likely to develop NDDs (Morillo and Guzmán, 2018; Carlos Oliva et al., 2020; Ron Benavides, 2021). This may be due to the conditions of chronic stress, exposure to violence, malnutrition, and lack of resources to which these children are frequently exposed.

In relation to this, it is essential to specify that NDDs are a set of alterations or problems present since early childhood that affect brain development and maturation, and which hinder the acquisition of motor, cognitive, emotional and social skills, being one of the most frequent causes of school failure. Their origin is multifactorial, and they are influenced by genetic and environmental factors (López and Förster, 2022). Among the most common NDDs we can find Attention Deficit and Hyperactivity Disorder (ADHD), one of the most prevalent disorders in childhood (Faraone et al., 2003), characterized by isolated or combined difficulties with attention, impulsivity or hyperactivity, beginning before the age of 12 (American Psychological Association, 2014). Other characteristics very commonly found in ADHD patients can be excessive impatience, mood swings, low self-esteem, sensitivity to rejection, stress, anxiety, trouble overcoming setbacks, procrastination, poor emotional control, or overwhelming feelings (Sapkale and Sawal, 2023).

Specific Learning Disorders (SLD) are neurodevelopmental disorders which involve difficulties in the acquisition and use of one or more skills important for learning. These difficulties often first become apparent in school, may persist into adulthood, and potentially lead to performance significantly below the level expected for the child’s intellectual ability, age and education (American Psychological Association, 2014). They may manifest as: Slow or imprecise reading and difficulty in reading comprehension (dyslexia), orthographical and written expression difficulties (dysgraphia), and difficulties in acquisition of numerical sense, mathematical reasoning, calculation, and arithmetic (dyscalculia) (Velasco González et al., 2022). In addition, within NDDs, Autism Spectrum Disorders (ASD) are also frequent, characterized by a primary affectation of communication (verbal and non-verbal) and difficulties in social interaction, along with the presence of restricted interests, rigid patterns of behavior, stereotyped behaviors and reactivity to sensory stimuli (American Psychological Association, 2014). When talking about social cognition difficulties, we find that people with ASD have major difficulties in their development of emotion recognition, theory of mind and social attention (Happé et al., 2017). Asperger’s Syndrome is currently included among the mild manifestations of ASD (grade 1, without associated intellectual disability).

Considering the above, it is of great importance to determine the interventions that can have positive effects on the cognitive development of children with NDDs, especially those living in disadvantaged socioeconomic conditions that exacerbate their consequences. Some research has demonstrated the effectiveness of neuropsychological stimulation programs in improving the cognitive, social, and emotional functioning of minors, due to their ability to promote brain plasticity and generate changes in brain functioning and structure (Espert Tortajada and Villalba Agustín, 2014). In addition, this research has shown that these programs may have greater benefits in populations of low socioeconomic strata (Company-Córdoba et al., 2021a), however research focusing on this specific population is sparse.

Therefore, the principal objective of the present study was to determine the impact of a cognitive stimulation program on four fifth-grade children located in vulnerable areas of Guatemala and at risk of exclusion (each with a different NDD), compared with a reference neurotypical sample. It was hypothesized that such a cognitive stimulation program carried out in a group environment would be beneficial for the cognitive development of all these minors at risk of social exclusion, being especially stimulating for those minors with NDDs given their greater needs for specific educational attention.

## 2 Method

### 2.1 Participants

The schools which the recruited participants attended were located in vulnerable areas in the suburbs of Guatemala City, characterized by high levels of poverty and exposure to violence (Rodríguez-Prieto et al., 2024). The neurotypical children who participated in this study (*n* = 126) had the following inclusion criteria:

- 5th grade students with no psychological, neurological, or neuropsychological clinical history.
- Not have repeated any school year.
- Have a reading and nonverbal intelligence performance in the screening tasks (L-3-DEs and TONI-2) above the 5th percentile.

The neurotypical children were randomly distributed in two groups: (1) the experimental group (*n* = 66), which received the cognitive stimulation program, and (2) the control group (*n* = 60), which carried out habitual reading activities during the intervention time. These would be the reference groups for comparison with the selected cases, as 2 of them were included in the experimental group and the other 2 on the control group. The neurotypical children were recruited from the same educational centers to serve as reference groups, allowing us to compare the results of the four selected cases of NDDs with the results of reference groups matched by grade, school, socioeconomic context, and type of intervention. The 4 selected cases were Spanish-speaking non-bilingual children recruited from the same school population as the neurotypical children. Only information on their primary guardian could be collected. They showed the following specific characteristics:

**Case 1**: 11-year-old male, who participated in the experimental group. He had a diagnosis of SLD with difficulty in reading (dyslexia) and written expression (dysgraphia). No difficulties were reported during pregnancy, but some cognitive difficulties were reported during the child’s language development. His primary guardian had 11 years of schooling and was a housekeeper. A monthly household income of 3,200 Guatemalan Quetzals (GTQ), which corresponds to approximately 375 euros, was reported. According to the Latin American and Caribbean Food Security Scale (ELCSA), the household lived in conditions of severe food insecurity (Score: 11).

**Case 2**: 14-year-old female, who received the stimulation program of the experimental group. Diagnosed with ASD, Asperger’s type. No perinatal problems or other difficulties in the child’s development were reported. Her mother had 13 years of schooling and an income of 1,000 GTQ per month in the family unit was reported (≈ 117€). This household was classified as having severe food insecurity (a score of 14 on the ELCSA).

**Case 3**: 11-year-old male, belonging to the control group. He presented with a diagnosis of ADHD, with no information on pharmacological treatment. No perinatal problems were reported. His mother was a housewife with 9 years of schooling and reported a monthly income of 3,200 GTQ (≈ 375€). This household had moderate food insecurity (a score of 7 on the ELCSA).

**Case 4**: 12-year-old male, belonging to the control group. He had a diagnosis of SLD, specifically, a difficulty in reading (dyslexia). No perinatal problems were reported, but language problems were reported during the child’s development. His mother was a housekeeper with 9 years of schooling, reporting a monthly household income of 800 GTQ, (≈ 93€). The household was living in conditions of severe food insecurity (a score of 13 on the ELCSA).

**Neurotypical group**: This group was comprised of 126 minors (42.4% female) aged between 10 and 11 years (*X* = 10.9, *SD* = 0.23), mostly non-bilingual Spanish speakers (98.4%), with no report of previous clinical problems. The legal guardians of this group had an average of 9 years of schooling (*X* = 9.1, *SD* = 3.7), with an average monthly income of 3,103 GTQ (*SD* = 1717.5) (≈ 364€). In relation to food security, 62.4% were in conditions of mild insecurity, 24% were moderately insecure and 13.6% were severely insecure.

### 2.2 Instruments

Prior to the commencement of the study, socio-economic, linguistic, and clinical questionnaires were administered to the families with the aid of the teachers. Subsequently, for both the pre (baseline) and post intervention assessments, a comprehensive neuropsychological battery was administered to all participants, requiring three 45-min sessions to complete for both the baseline and post assessments (see detailed description in the Supplementary Appendix 1):

- Language:
  - *TOKEN Test*, verbal comprehension (De Renzi and Faglioni, 1978; Olabarrieta-Landa et al., 2017)
  - *Test of verbal fluency*, phonological and semantic (Portellano et al., 2009)
  - *Inter-American Reading series*, vocabulary, Speed, and Comprehension (L-3-DEs) (Herschel, 1962)
- Attention and Executive Functions:
  - *Nesplora Aula School*, attention, inhibition, impulsivity, and speed of response (Climent Martinez, and Banterla borzaga, flavio, 2016)
  - *Nesplora Ice Cream*, working memory, flexibility and planning (Climent Martínez et al., 2021)
  - *Test of Nonverbal Intelligence, TONI-2* (Brown et al., 1990/2001)
- Social Cognition
  - *Neuropsychological Evaluation Battery NESPY-II* (Korkman et al., 2014): subtests of Emotion Recognition and Theory of Mind.

**Figure 1:**
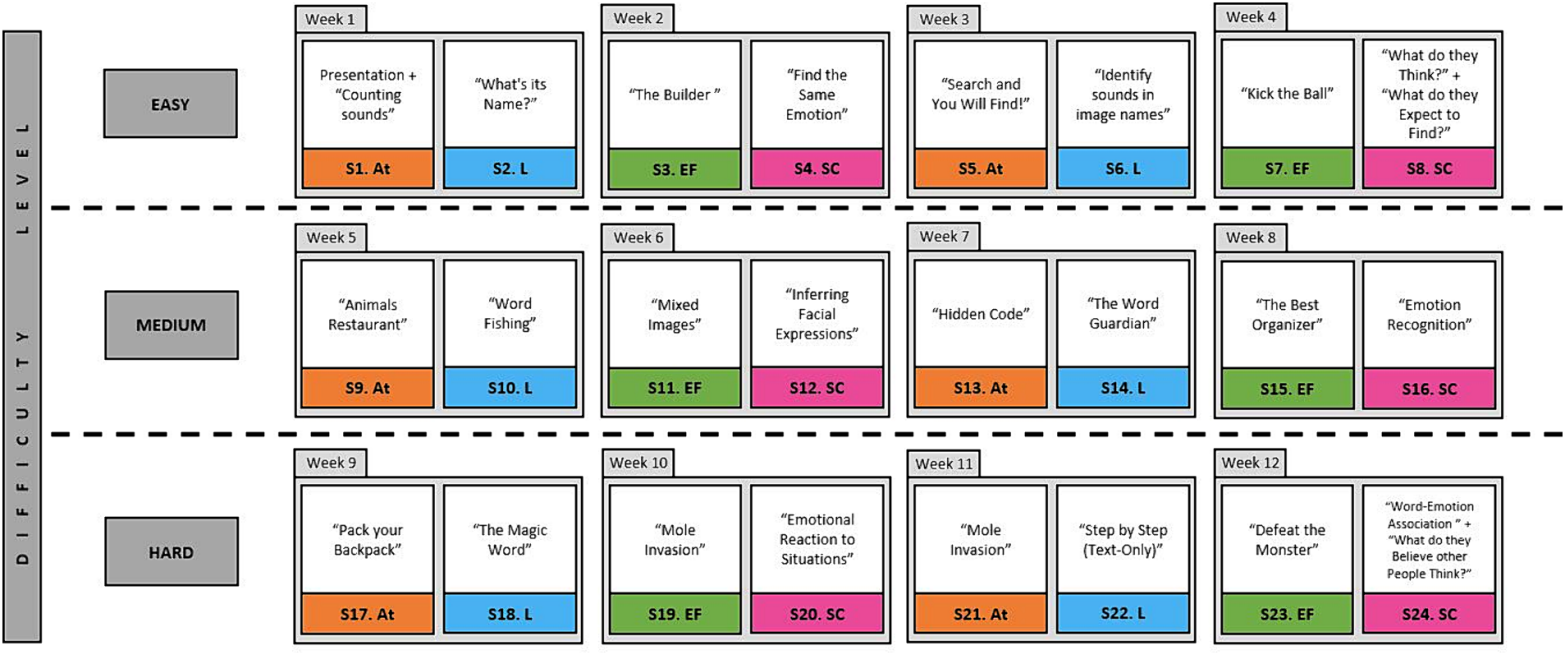
Integral cognitive stimulation program session by session.

The intervention was implemented via the use of tablets, using a cognitive and emotional stimulation protocol designed by specialists in neuropsychology, and accessed through the NeuronUP platform (Rodríguez-Prieto et al., 2024). NeuronUp is a digital tool, with ecological validity for cognitive rehabilitation and stimulation, which allows managing interventions, users and results in a personalized way (Neuron UP, 2021). The applied program focused on the stimulation of four cognitive domains: attention, language, executive functions, and social cognition. It was structured in 24 sessions divided into three levels of increasing difficulty (see Figure 1). Both groups used tablets during the development of the study to control possible biases in the results derived from the use of digital devices.

### 2.3 Procedure

The present study received the approval of the Ethics Committee of the Faculty of Social Sciences of the Universidad del Valle de Guatemala (Protocol approval #56 of May 31, 2021). It did not involve any risks for the participants and was developed following the recognized international guidelines and regulations of the Declaration of Helsinki.

The initial assessment was carried out in the participants’ own educational centers by four trained professionals. Best efforts were made to carry out the assessments in a comfortable, quiet room, but conditions varied between schools. Consequently, acoustic and visual distractions were unavoidable in some locations. Once the first assessment was completed, each group (both the cognitive stimulation group and the reading group) received two 30-min sessions per week on alternate days for 12 weeks (24 sessions in total), conducted in a group setting. A post-intervention cognitive evaluation of the participants was carried out 1 week after the final intervention session to determine the impact of the intervention program on the children.

### 2.4 Data analysis

To establish intrasubject clinically significant differences, a comparison was made between the pre- and post-intervention percentile scores. The changes observed for each of the four cases are described using qualitative descriptions based on Korkman et al. (2014) (see the Supplementary materials for further details).

Next, to have a fully quantitative measure for analysis, the delta values for all participants were determined by calculating the difference between pre- and post-intervention percentile scores from the neuropsychological test variables. The delta scores for the four cases studied were then compared with the delta scores of the matched reference groups using one standard deviation (*SD*) as an indicator of significant differences (see Supplementary Appendix 2, for a detailed description of the criteria followed for the clinical interpretation of percentile scores and *SD*).

## 3 Results

### 3.1 Analysis of intrasubject changes in clinical significance

Comparisons between pre- and post-intervention percentile scores in the domains of Language, Attention, Executive Functions, and Social Cognition are shown in Table 1. For a better understanding of the following descriptions, we recommend following Table 1 as a visual reference. In general, there were more clinically significant increases in the cognitive performance of the 2 cases who participated in the experimental group of cognitive stimulation, than those recorded by the 2 cases who participated in the reading control group. Specifically, the cases in the experimental group improved on 8 and 5 cognitive measures, respectively, while the cases in the control group only improved on 3 measures each. It is also important to note that in some domains, a decrease was seen between pre- and post-scores. However, the 2 cases in the experimental group showed a decrease in just one category each, and this was only of one level of clinical significance. In contrast, the 2 cases who participated in the control group showed decreases in 2 and 4 categories, respectively, and some of these decreases were more than one level of clinical significance.

**Table 1:** Pre and post assessment percentile scores (Pc) of the case studies.

	Case 1		Case 2		Case 3		Case 4	
	Stimulation group (Experimental)				Reading group (Control)			
	Pc Pre	Pc Post	Pc Pre	Pc Post	Pc Pre	Pc Post	Pc Pre	Pc Post
*Language*								
Language comprehension	30	10^†^	1	5	20	90^*^	1	1
Phonological fluency	0.1	1^*^	0.1	1^*^	0.1	1^*^	0.1	0.1
Semantic fluency	60	80	70	30	95	95	30	10^†^
Reading skills	35	75	97	99	80	90^*^	5	25^**^
*Attention and executive functions*								
Attention	7	26^*^	1	4	59	25	8	2^†^
Inhibitory control	3	13^*^	15	30^*^	66	6^†^	20	2^††^
Impulsivity	2	47^**^	62	87^*^	65	29	31	11^†^
Response speed	11	32^*^	26	33	35	44	14	20
Working memory	21	21	30	76	34	42	1	14^*^
Cognitive flexibility	88	42^*^	51	64	51	27	64	60
Planning	12	59^*^	11	38^*^	23	69	39	44
Nonverbal intelligence	17	50	94	62^†^	17	83	11	6
*Social cognition*								
Emotion recognition	9	25^*^	50	75	37	2^††^	0.1	9^**^
Theory of mind	38	63	8	38^*^	76	63	1	1

^*^Clinically significant increase of 1 level.

^**^Clinically significant increase > 1 level.

^†^Clinically significant decrease of 1 level.

^†^^†^Clinically significant decrease > 1 level.

Looking in more detail at case 1 (SLD), it can be seen from Table 1 that this child showed clinically significant increases in 8 measures: phonological fluency, attention, inhibitory control, impulsivity, response speed, cognitive flexibility, planning, and emotion recognition. In general, these improvements were from a medium-low or low performance (corresponding to a clinical significance of mild deficit) to a medium performance (considered to be clinically normal). The only category in which this child maintained a low performance post intervention was in the category of phonological fluency, although a significant improvement was also observed in this category. Whilst her performance in working memory remained stable, a decrease in language comprehension was observed, with the score falling from normal performance to a slightly deficient performance. The other child in the experiment group, case 2 (ASD), showed clinically significant increases in the following 5 measures: phonological fluency, inhibitory control, impulsivity, planning, and theory of mind. While in the phonological fluency measure, he improved from a severe to a moderate deficit level, in the inhibitory control measure his post intervention performance reached the normal range. Similarly, on the planning and theory of mind variables, his performance improved from a mild deficit to normal performance. Additionally, his performance in impulsivity improved to above average. The only measure in which his performance decreased significantly was in nonverbal intelligence, where, in any case, his score remained in what is considered the normal range.

Turning now to the two cases with NDDs who participated in the control group, case 3 (ADHD) exhibited increases of clinical significance in 3 measures: language comprehension, phonological fluency and reading skills. Specifically, this participant’s post-intervention comprehension and reading scores fell within the “talented” range, while phonological fluency improved from severe to moderate deficit. This child maintained a stable performance in semantic fluency and presented a decrease of clinical significance in 2 measures: inhibitory control and emotion recognition, dropping from normal performance to mild deficit and moderate deficit, respectively. Case 4 (SLD) also showed a significant increase in 3 measures: reading skills (from moderate deficit to normal performance), working memory, and emotion recognition (from moderate and severe deficits, respectively, to mild deficits). However, this child exhibited decreases in his level of clinical significance in 4 measures, the most decreases exhibited by any of the 4 cases. Specifically, semantic fluency, inhibitory control, and impulsivity, all dropped from normal performance to a mild or moderate deficit performance, while attention went from a mild to a moderate deficit.

### 3.2 Analysis of changes in cognitive performance compared to reference groups

**Figure 2:**
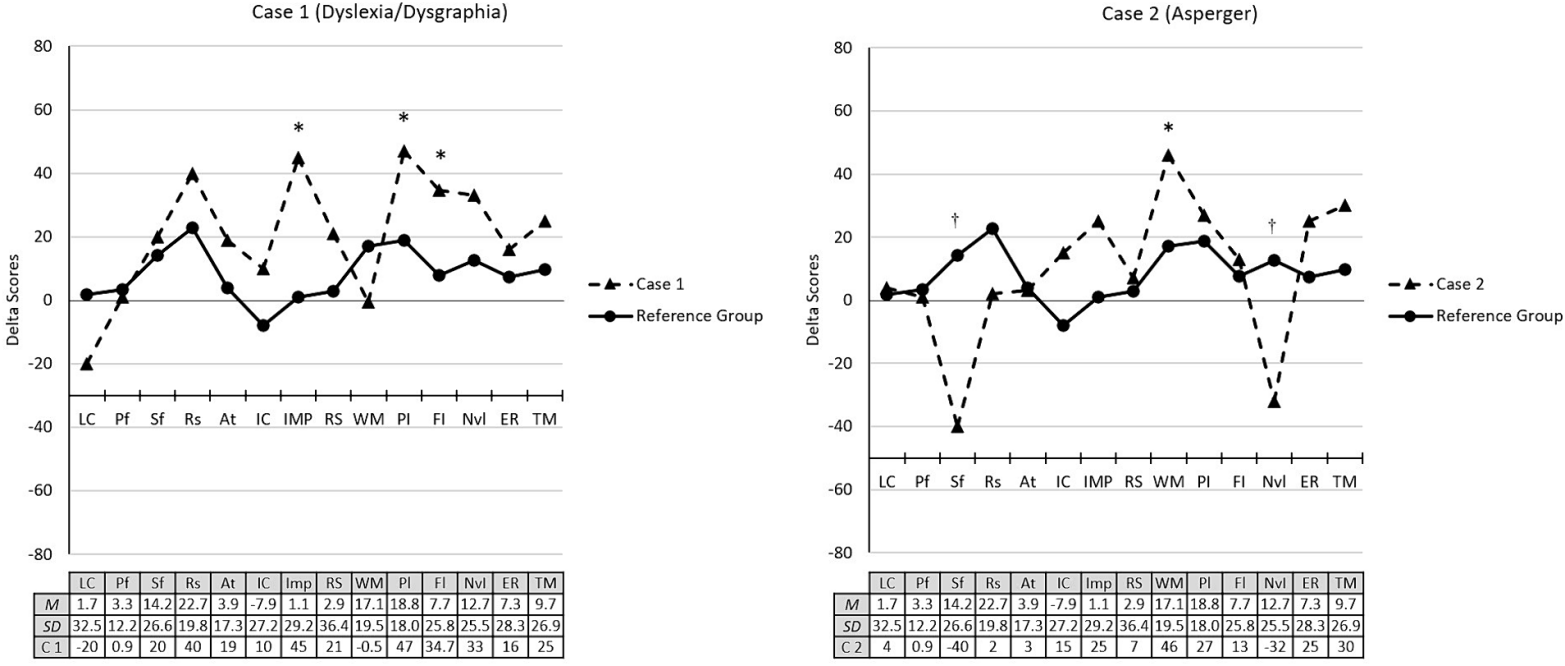
Change in pre/post cognitive performance (standardized delta scores) of cases 1 and 2 compared to their reference group (experimental). LC, Language comprehension; Pf, Phonological fluency; Sf, Semantic fluency; Rs, Reading skills; At, Attention; IC, Inhibitory control; IMP, Impulsivity; RS, Response speed; WM, Working memory; Pl, Planification; Fl, Flexibility; Nvl, Non-verbal Intelligence; ER, Emotion recognition; TM, Theory of mind. *Indicates a clinically significant increase in the scores. ^†^Indicates a clinically significant decrease in the scores.

To perform a more quantitative analysis of pre/post differences, changes in the delta scores were contrasted between the 4 NDD cases and their corresponding reference groups (experimental or control). To achieve this, the means and *SD*s of all delta scores for all measures were calculated for the control and experimental groups. The delta scores for each of the four cases were then converted into *SD* units by comparing them to the relevant *SD* values from their corresponding reference group. Based on a threshold of 1 *SD* (see Supplementary materials), the case 1 child (SLD) showed a significant improvement in measures of impulsivity (1.5 *SD*), cognitive flexibility (1 *SD*), and planning (1.6 *SD*) (see Figure 2). Similarly, the child with ASD (case 2) showed a significantly higher delta score (1.5 *SD*) on the working memory measure, compared to the experimental group. However, in relation to the measures of semantic fluency and nonverbal intelligence, the effect was significantly smaller, as the delta scores on these measures were found to be one *SD* below the mean of the normative group.

**Figure 3:**
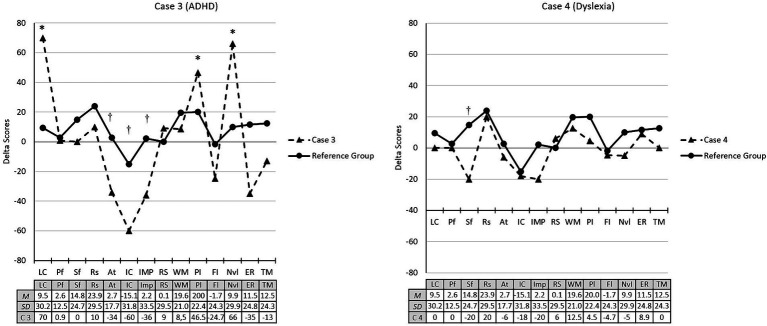
Change in in pre/post cognitive performance (standardized delta scores) of cases 3 and 4 compared to their reference group (control). LC, Language comprehension; Pf, Phonological fluency; Sf, Semantic fluency; Rs, Reading skills; At, Attention; IC, Inhibitory control; IMP, Impulsivity; RS, Response speed; WM, Working memory; Pl, Planification; Fl, Flexibility; Nvl, Non-verbal Intelligence; ER, Emotion recognition; TM, Theory of mind. *Indicates a clinically significant increase in the scores. ^†^Indicates a clinically significant decrease in the scores.

Regarding the impact of the reading program (control group), in case 3 (ADHD) a greater increase in this child’s delta scores for language comprehension (2 *SD*), planning (1.2 *SD*) and nonverbal intelligence (1.9 *SD*) was observed, compared to their reference group (see Figure 3). However, the intervention effect was smaller than in their reference group for attention (−2 *SD*), inhibitory control (−1.4 *SD*), and impulsivity (−1.1 *SD*). Finally, case 4 (SLD, dyslexia) did not show significant differences with the scores obtained by his reference group, except for the semantic fluency measure (−1.4 *SD*), in which he showed a significant drop in performance with respect to his group.

## 4 Discussion

The main objective of this study was to analyze the effect of a cognitive stimulation program in a series of cases of children with NDDs from disadvantaged socioeconomic strata in Guatemala and compare their results with those obtained normative reference groups. In accordance with our expectations, significant performance improvements were observed after the implementation of the experimental cognitive stimulation program. These improvements were mainly related to executive functions and social cognition, agreeing with previous studies which have demonstrated the effectiveness of cognitive stimulation programs in improving cognitive, emotional and social functioning of children (Ministerio de Sanidad – Política Social e Igualdad, 2010; Ghiglione et al., 2011; Espert Tortajada and Villalba Agustín, 2014; Company-Córdoba et al., 2021b).

For case 1 (SLD, dyslexia and dysgraphia), significant improvements, in terms of clinical relevance, were found in measures of phonological fluency, attention, speed of response, executive functions (cognitive and behavioral inhibition, flexibility and planning), and emotion recognition. When these performance improvements were compared with those obtained by the normative group that also underwent the cognitive stimulation program, a significantly greater improvement was found on measures of behavioral control (impulsivity), cognitive flexibility, and planning, suggesting that the cognitive stimulation program had a greater positive effect on the performance of the executive functions of this case with NDD than in the normative developmental group.

In the second case (ASD), from a clinical relevance perspective, the results showed that the child improved her performance after the stimulation program in phonological fluency, inhibitory control, impulsivity, planning, and theory of mind. Furthermore, although her performance on the non-verbal intelligence task decreased, it remained within the normal range. When comparing her improvements with those of the rest of the normative experimental group, this child registered a significantly greater improvement in working memory than that shown by their reference group. Although her performance declined in semantic fluency and nonverbal intelligence, her post-intervention level is still considered to be in the normal range. This result could be explained by the difficult assessment conditions interfering with the participant’s performance. An important aspect of this case, given its relevance to ASD, is her clinically significant improvement in theory of mind following the stimulation program. This result suggests that this child improved her abilities to understand the emotions, intentions, and thoughts of others (Korkman et al., 2014). Thus, although the program did not result in an improvement in a wider range of skills for this participant with respect to her reference group, taking into account her ASD condition, the improvements she showed can already be considered an important benefit of the stimulation program. This is because an improvement in skills that contribute understanding and implementing effective social interactions significantly contribute to the quality of life of people with ASD (Pérez Rivero and Martínez Garrido, 2014).

As for the cases with NDDs who participated in the control group reading activity, the results of case 3 (ADHD) showed an improvement in language comprehension, phonological fluency, and reading skills. This suggests that the reading program is also a stimulating action for the development of language and reading. However, when other cognitive functions were analyzed, a performance decrease was observed in the inhibitory control and emotion recognition at the end of the program. Furthermore, when comparing the development of these skills with the reference control group, despite showing a greater development in language comprehension, planning, and nonverbal intelligence, a lower development in some of the skills that are commonly affected in ADHD cases, such as attention, inhibitory control and impulsivity was observed. This suggests that, although the reading program could be beneficial for the development of some cognitive functions (especially linguistic), it would not be sufficient to stimulate the development of these functions, which are especially deficient in ADHD (American Psychological Association, 2014). Finally, the results obtained by the child in the fourth case (SLD, dyslexia), showed an improvement in his reading performance and working memory after the reading program, as well as an improvement in his ability to recognize emotions. This again supports the benefits that this type of program can have on reading and related skills (Nevo et al., 2016; Carretti et al., 2017; Gao et al., 2018). However, in this case, there was also a significant decrease in performance in semantic fluency, attention, inhibitory control, and impulsivity, skills that were not specifically stimulated by belonging to the control group. When comparing the pre-post changes of this case with the changes shown by the reference group, semantic fluency was the only area with very different outcomes, the control group improved between the pre and post evaluations while the child with dyslexia declined. These results would allow us to reaffirm what was mentioned in the previous case, highlighting the utility of specific neuropsychological interventions in addition to the usual reading promotion programs to promote the overall performance of children with NDDs.

By comparing the four case studies we can obtain a more global view of the effects of the neuropsychological intervention program versus the reading program. The results showed that in the two cases that received the cognitive stimulation program, more delta scores were found which were equal to or above those of their reference group (the experimental group). This contrasts with the results of the two cases that completed the control reading program, where delta scores tended to be equal to or below those of their reference group (the control group). This seems to indicate that the computerized neuropsychological intervention would be even more effective for children diagnosed with NDDs than for normative children from socioeconomically disadvantaged areas. Literature regarding interventions with children diagnosed with NDDs living in these conditions is difficult to find, however, there are plenty of studies supporting this kind of intervention for population with NDDs from less disadvantaged contexts (Oldrati et al., 2020; Ren et al., 2023). On the other hand, it is possible to conclude that the reading program was a good strategy for the development of language and reading skills, but it was less stimulating for the development of other cognitive functions relevant to cases with NDDs. As a final observation, we have to mention that our goal in this study was to implement a program applicable to entire groups. That is, a program that can be applied in a group setting in order to minimize the resources required, together with reaching a large number of children quickly. This may mean that the program could be less effective and not be as adaptable as individually designed interventions. However, taking into account the target population, that is children living in socioeconomically disadvantaged conditions, and due to the potential lack of resources in these communities, we believe that in many cases it would be more feasible to implement a group based intervention program rather than trying to create individualized programs, as the benefit they can derive from these interventions is still significant (Renou and Doyen, 2019).

One of the main methodological limitations was to have only four cases, which means the results should be interpreted with caution. Other limitations were the environmental factors that were difficult to control in the participating schools, such as background noise and cramped testing spaces. Similarly, due to the lack of normative scores and previous research undertaken with Central American populations, some Spanish norms had to be used as reference. However, the impact of is problem was minimized since the analyses were carried out with an intrasubject design. Finally, future studies should evaluate a larger number of cases in order to try and compare equally matched sample sizes for NDDs and healthy controls which have been matched by age, gender and socioeconomical characteristics.

## 5 Conclusion

This study contributes to the knowledge on the effectiveness of cognitive stimulation programs in promoting the development of children with a high degree of clinical and psychosocial vulnerability, such as those who present NDDs and belong to disadvantaged socioeconomic contexts. As far we know, few neuropsychological studies have focused their attention on the population with these two conditions, so it is essential to present evidence of the effectiveness of these programs and the need to design them at affordable costs for people with limited resources. The main conclusions of this study are:

- The cognitive stimulation program resulted in a significant improvement in the executive functions and social cognition (emotion recognition and theory of mind) of the children with neurodevelopmental disorder, which is of great importance considering that it allows them to improve their behavioral self-regulation skills and increase their understanding of the social world around them in order to confront the challenges they face.
- Although the reading promotion program implemented in the control group had positive effects on the reading ability of the children, it did not influence other cognitive components and its effect was apparently lower in children with neurodevelopmental disorders compared to their reference group.
- The results show that the cognitive stimulation program had a greater positive effect in the cases with neurodevelopmental disorders than in the normative experimental group, indicating that this type of specialized interventions is especially effective in a population with clinical characteristics, making it possible to shorten the gap in their development with respect to their reference group.

## Data availability statement

The raw data supporting the conclusions of this article will be made available by the authors, without undue reservation.

## Ethics statement

The studies involving humans were approved by Ethics committee of the Faculty of Social Sciences from Universidad del Valle of Guatemala. The studies were conducted in accordance with the local legislation and institutional requirements. Written informed consent for participation in this study was provided by the participants’ legal guardians/next of kin. Written informed consent was obtained from the minor(s)’ legal guardian/next of kin for the publication of any potentially identifiable images or data included in this article.

## Author contributions

PR-P: Data curation, Formal analysis, Investigation, Project administration, Software, Visualization, Writing – original draft. NG-O: Formal analysis, Visualization, Writing – original draft. IS: Conceptualization, Formal analysis, Methodology, Visualization, Writing – review & editing. JI-A: Conceptualization, Funding acquisition, Methodology, Project administration, Resources, Supervision, Visualization, Writing – review & editing.
