# Supplementary material for: Cognitive stimulation in socioeconomically disadvantaged children with neurodevelopmental disorders: a case series
Source: Front Psychol. 2024 Jul 9;15:1365697. doi: 10.3389/fpsyg.2024.1365697 (PMC11263084; doi:10.3389/fpsyg.2024.1365697)
Supplement: Supplementary file 1 [file Table_1.DOCX]

**Cognitive Stimulation in Socioeconomically Disadvantaged Children with Neurodevelopmental Disorders: A case series.**

**Supplementary material**

**Appendix 1**

*Neuropsychological assessment tests*

- TOKEN Test, reduced version (de Renzi & Faglioni, 1978; Olabarrieta-Landa et al., 2017). It measures the capacity to understand language in an order without redundancy. In this task, a series of shapes (big and small circles and squares of various colours) are laid out on the table in front of the participant, and then they are told to touch or move them providing increasingly complicated instructions. In its reduced version it has 36 items and has a duration of about 10 minutes, with a maximum score of 36 and a minimum of 0.
- Verbal fluency test (Portellano et al., 2009). It measures phonological (words starting with “M”) and semantic (words included in the category “animals”) verbal fluency through the number of words produced by the person each time (1 minute per condition). It takes about 4 minutes to complete. These tasks have a minimum score of 0 but don’t have an stablished maximum score because it depends on how many words can a person produce in 1 minute.
- Inter-American Reading Series (L-3-DEs) (Herschel, 1962). This is an assessment task that can be administered in groups and measures reading proficiency of children, having subscales for vocabulary, comprehension speed and comprehension level. The version used in this study is a commercial version culturally and linguistically adapted for the Guatemalan population. It has an average duration of 60 minutes and has a maximum score of 125 and a minimum of 0. The scores of this task are used as a screening for inclusion criteria. The participants needed to have at least a very basic ability to read and understand instructions for the program to influence them.
- Nesplora Aula School (Climent-Martínez & Bánterla-Borzaga, 2016). A virtual reality test for the evaluation of several abilities related to attention and self-regulation in children with an ecological approach. In this task, the participants are sitting in a classroom, and they have to pay attention to the blackboard and the teacher. The task presents various stimuli and tells them which ones they must react to and which ones they must ignore. The set of instructions changes throughout the task. In this project we used these main variables: 1) Attention (total omission mistakes), 2) Inhibitory Control (total commission mistakes), 3) Impulsivity (mean reaction time while committing mistakes), and 4) Response speed (mean reaction time when emitting correct responses). It has an average duration of 15 minutes, and the results are given directly as percentile scores depending on the participant’s age. It has an average duration of 15 minutes, and the results are given directly as percentiles scores depending on the participant’s age.
- Nesplora Ice Cream (Climent-Martínez et al., 2021). A virtual reality test for the evaluation of Executive Functions. In this task, people have to attend customers in an ice cream shop, giving priority to the orders depending on how the customer is dressed. Customers order different flavors and it is up to the participant to give them the correct one from a recipe book that changes mid-task. We used the main variables: 1) Working Memory (mean between total amount of correct responses and total processing speed), 2) Planification (mean between number of turns assigned and the average round duration), and 3) Cognitive Flexibility (mean between the task interference -difference between correct responses in the two halves of the task-, perseverative behaviour -number of incorrect responses during the second half of the task, which were correct in the first half-, and switching -time increment between turns-). It has an average duration of 30 minutes, and the results are given directly as percentiles scores depending on the participant’s age.
- Non-verbal Intelligence Test (TONI-2) (Brown et al., 1990; de la Cruz, 1995). It was developed as an intelligence measurement free of language influence, motor skills or reading capabilities, looking only for reasoning skills to solve abstract puzzles. It consists of 55 items of increasing difficulty, lasting approximately between 15-20 minutes. It has a maximum score of 55 and a minimum of 0. The scores of this task is also used as a screening for inclusion criteria.
- NESPY-II Assessment Battery (Korkman et al., 2007/2014). This exhaustive neuropsychological battery is designed for the cognitive assessment of children and adolescents. In this project we used two subscales directly related to Social Cognition skills: 1) Emotion Recognition, and 2) Theory of Mind. The task Emotion Recognition measures the ability of children to correctly identify emotions in pictures of other children. It has 35 items and has a duration of about 5 minutes, with a maximum score of 35 and a minimum of 0. The Theory of mind task measures the ability of children to interpret and understand beliefs, intentions, faking and lies, emotions, and imagination in other people. It also measures if kids can understand social context and its relationship with emotions. It consists of 21 items and has a duration of about 5 minutes, with a maximum score of 28 and a minimum of 0.

*Socio-economic, linguistic and clinical assessment*

- A sociodemographic questionnaire was handed to the families to give out the following information: names, age, occupation, and mean educational level of legal guardians (MLPE), together with monthly income of the family household in Quetzals (Q).
- A clinical questionnaire will be administered asking if there were any health problems for the mother or child during pregnancy or childbirth, if the child needed to be in an incubator, if the child had any clinical diagnostic, and if it was or is currently under any pharmacological treatment.
- A language experience questionnaire that will assess the languages that both the legal guardians and the children can understand and speak, including their degree of proficiency.
- The Latin-American and Caribbean Latin-American Food Security Scale (ELCSA)(Ballard et al., 2013), validated by the Food and Agriculture Organization of the United Nations. This scale is composed of two different parts answered by the caregivers. First, caregivers are asked how safe they feel in relation to the food they can provide to the family. The assessment of this part offered a score between 1 to 15 in which higher scores reflect higher food insecurity. The second section asks how many days per week the family consumes a certain type of food like vegetables, meat and fish, sugary foods, etc.

**Appendix 2**

*Changes in clinical significance*

The analysis of the pre- and post-intervention results was based on clinically significant differences, determined by a change in the participant's clinical classification following Korkman et al. (2007/2014) recommendations. The clinical ratings can be seen in the *Description* and *Clinical Significance* columns in Table S1. The Description values refer to qualitative categories indicating the participant's performance compared to the rest of the population. Starting at PC 50 (average), a series of 3 improvements, each of magnitude 1SD, would see the child reclassified as "medium-high", "high" and "very high", respectively. Similarly, a series of 3 reductions in performance, each of magnitude 1SD, would see the child reclassified as "medium-low", "low" and "very low", respectively. For this reason, when comparing the cases to their reference groups, we decided to use an improvement of 1SD (above or below that demonstrated by the reference group) as marking a significant (i.e., practical) improvement in the performance of the child. The Clinical Significance column indicates the interpretation that professionals may attribute to different levels of performance, depending on whether they are considered gifted, normal, or deficient; and to what degree they would deviate from normal performance.

Table S1. Qualitative description and clinical significance of percentile scores (PC) and standard deviations (SD). Based on Korkman et al. (2007/2014).

| PC (range) | *SD* | Description | Clinical significance |
| --- | --- | --- | --- |
| 99,9 | 3,0 |  |  |
| 99,6 | 2,7 | Very High | Extremely talented |
| 98-99 | 2,3 |  |  |
| 95-97 | 2,0 |  | Highly talented |
| 90-94 | 1,7 | High |  |
| 85-89 | 1,3 |  | Talented |
| 76-84 | 1,0 | Medium-High |  |
| 63-75 | 0,7 |  |  |
| 51-62 | 0,3 |  |  |
| 50 | 0 | Average | Normal |
| 38-49 | -0,3 |  |  |
| 26-37 | -0,7 |  |  |
| 17-25 | -1,0 | Medium-Low |  |
| 11-16 | -1,3 |  | Small deficit |
| 6-10 | -1,7 | Low |  |
| 3-5 | -2,0 |  | Moderate deficit |
| 1-2 | -2,3 |  |  |
| 0,4 | -2,7 | Very low | Severe deficit |
| 0,1 | -3,0 |  |  |
| Note. PC = Percentile, *SD* = Standard Deviation | | | |
